# Quantum *κ*-Entropy: A Quantum Computational Approach

**DOI:** 10.3390/e27050482

**Published:** 2025-04-29

**Authors:** Demosthenes Ellinas, Giorgio Kaniadakis

**Affiliations:** 1 School of ECE QLab, Technical University of Crete, 731 00 Chania, Greece; 2 Dipartimento di Scienza Applicata e Tecnologia, Politecnico di Torino, Corso Duca degli Abruzzi 24, 10129 Torino, Italy; giorgio.kaniadakis@polito.it; 3 Istituto dei Sistemi Complessi, Consiglio Nazionale di Ricerca, 00185 Rome, Italy; 4 Sezione di Torino, Istituto Nazionale di Fisica Nucleare, 10125 Torino, Italy

**Keywords:** κ-Entropy, quantum computation, quantum information

## Abstract

A novel approach to the quantum version of κ-entropy that incorporates it into the conceptual, mathematical and operational framework of quantum computation is put forward. Various alternative expressions stemming from its definition emphasizing computational and algorithmic aspects are worked out: First, for the case of canonical Gibbs states, it is shown that κ-entropy is cast in the form of an expectation value for an observable that is determined. Also, an operational method named “the two-temperatures protocol” is introduced that provides a way to obtain the κ-entropy in terms of the partition functions of two auxiliary Gibbs states with temperatures κ-shifted above, the hot-system, and κ-shifted below, the cold-system, with respect to the original system temperature. That protocol provides physical procedures for evaluating entropy for any κ. Second, two novel additional ways of expressing the κ-entropy are further introduced. One determined by a non-negativity definite quantum channel, with Kraus-like operator sum representation and its extension to a unitary dilation via a qubit ancilla. Another given as a simulation of the κ-entropy via the quantum circuit of a generalized version of the Hadamard test. Third, a simple inter-relation of the von Neumann entropy and the quantum κ-entropy is worked out and a bound of their difference is evaluated and interpreted. Also the effect on the κ-entropy of quantum noise, implemented as a random unitary quantum channel acting in the system’s density matrix, is addressed and a bound on the entropy, depending on the spectral properties of the noisy channel and the system’s density matrix, is evaluated. The results obtained amount to a quantum computational tool-box for the κ-entropy that enhances its applicability in practical problems.

## 1. Introduction

The κ-entropy introduced two decades ago in the trilogy of papers [1,2,3] assumes the form

(1) $$S_{\kappa }=\frac{1}{2\kappa }\sum_{i=1}^{W}\;\left(\rho_{i}^{\;1-\kappa }-\rho_{i}^{\;1+\kappa }\right),$$

where $\rho =\{\rho_{i}\}$ is the probability density. The above entropy arises naturally in the context of Einstein’s special relativity and generates a self-consistent κ statistical mechanics, which turns out to be a relativistic extension of classical Boltzmann–Gibbs statistical mechanics. which is obtained in the κ→0 classical limit.

The persistent power-law tails of the cosmic rays spectrum, spanning 13 decades in terms of energy and 33 decades in terms of particle flux, turn out to be a purely relativistic effect correctly predicted by κ statistical mechanics and this result represents one of the greatest successes of the new theory.

The statistical theory based on $S_{\kappa }$ has an axiomatic structure and can also be introduced without reference to special relativity [4], since it also has applications outside of relativistic physics.

In the last two decades, many authors have studied the theoretical foundations of the underlying thermodynamics, [5,6,7,8,9,10,11,12,13] and the mathematical structure of the theory, c.f. [14,15,16,17,18,19,20,21,22,23,24,25,26].

On the other hand, specific applications of the theory have been considered in various areas of the science of complex physical, natural or artificial, classical or quantum systems. With regard to the applications concerning quantum systems, we recall the studies devoted in particular to quantum mechanics [27,28], quantum hadrodynamics [29], quantum statistical mechanics, c.f. [30,31,32], quantum gravity [33,34,35,36,37,38,39,40] and quantum cosmology, c.f. [41,42,43,44,45].

The content of the present paper frames the quantum version of κ-entropy and relates it to the conceptual, mathematical and operational framework of quantum computation. The relations developed are organized into three scopes, each one containing two propositions, that form the three main chapters of the paper, respectively. All proofs are deferred to a final Appendix A. The following outline describes the matter:

*Scope 1*: The aim is to consider a canonical Gibbs state density matrix for some Hamiltonian and determine an operational form for its kappa canonical quantum entropy via the expectation value of a quantum observable, Proposition 1; and further, to introduce a two-temperatures protocol for measuring the canonical state kappa entropy, Proposition 2.

*Scope 2*: The aim (channel generating kappa entropy) is to express the kappa entropy for a general density matrix via a positive and trace-preserving quantum channel as well as via its unitary dilation, Proposition 3; and further, to simulate the kappa entropy via a generalized form of the quantum circuit of the so-called Hadamard test, Proposition 4.

*Scope 3*: The aim is to relate the kappa and von Neumann quantum entropies between them and to determine the bounds on their difference, Proposition 5; and further, to examine the effect of a typical noise, i.e., a quantum random unitary channel acting on an original quantum system, by evaluating bounds on the value of the kappa entropy of the transformed density matrix. To gain full generality for the result, the bound is shown to be determined by the spectral properties of both the channel and systems’ density matrix, Proposition 6.

*Motivations:*κ-Entropy and its relations to other quantum entropies: Some of the motivation for the development of κ entropy in quantum information language is based on its relation to other standard quantum entropies, such as von Neumann (vNE) and Renyi entropy. Operational interpretations of vNE supporting its wide use in the quantum information field are well known, and two of them are invoked below. Here, they are useful in motivating a similar interpretation for the quantum κ entropy, c.f. Proposition 2 below. Similarly to vNE, the Renyi entropy shares common features with κ entropy, supporting the treatment of the latter in quantum language as outlined below.

*vN entropy intepretation 1*: Suppose that Alice prepares a quantum state ρ. Bob can then perform a particular POVM ${\left\{\Lambda_{x}\right\}}_{x\in X}$ to learn about the quantum system, where *X* denotes the random variable corresponding to the classical output of the POVM, i.e., $x\to \Lambda_{x}$. The probability density function $x\to p_{X}\left(x\right)$ of random variable *X* is then $p_{X}\left(x\right)=Tr\left(\rho \Lambda_{x}\right).$ The Shannon entropy of the POVM $\Lambda_{x}$ is denoted by $S_{sh}\left(X\right)=-\sum_{x\in X}p_{X}\left(x\right)\log\left(p_{X}\left(x\right)\right)=-\sum_{x\in X}Tr\left(\rho \Lambda_{x}\right)\log\left(Tr(\rho \Lambda_{x})\right).$ The minimum Shannon entropy over all rank-1 POVMs is equal to a quantity which is identified with the von Neumann entropy $S_{vN}\left(\rho \right)$ of the density operator ρ. Explicitly, this optimization means that $S_{vN}\left(\rho \right)=\min_{\left\{\Lambda_{x}\right\}}\left\{-\sum_{x\in X}Tr\left(\rho \Lambda_{x}\right)\log\left(Tr(\rho \Lambda_{x})\right)\right\},$ where the minimum is restricted to be over rank-1 POVMs, i.e., those with $\{\Lambda_{x}=|\psi_{x}\rangle \langle \psi_{x}{\left|\right\}}_{x\in X},$ satisfying $\sum_{x\in X}\Lambda_{x}=\sum_{x\in X}\left|\psi_{x}\rangle \langle \psi_{x}\right|=I,$ where the latter implies the completeness of states ${\left\{|\psi_{x}\rangle \right\}}_{x\in X}$ (p. 256, [46]).

*vN entropy intepretation 2*: Suppose that Alice generates a quantum state $|\psi_{x}\rangle$ in her lab according to some probability density $p_{X}\left(x\right)$ of a random variable *X*. Suppose further that Bob has not yet received the state from Alice and does not know which one she sent. The expected density operator from Bob’s point of view is then $\rho =E_{X}\left\{|\psi_{x}\rangle \langle \psi_{x}|\right\}=\sum_{x\in X}p_{X}\left(x\right)\left|\psi_{x}\rangle \langle \psi_{x}\right|.$ The interpretation of the entropy $S_{vN}\left(\rho \right)$ is that it quantifies Bob’s uncertainty about the state Alice sent—his expected information gain is $S_{vN}\left(\rho \right)$ qubits upon receiving and measuring the state that Alice sends (p. 254, [46]).

*Remark*: The development of κ entropy along the lines of quantum information put forward in this paper opens the possibility of using it to quantify, e.g., quantum entanglement, a procedure that has been carried out by other types of entropy, c.f. the entanglement Renyi α-entropy (ERαE) index, c.f. [47] and references therein. Below an outline of the important features of Renyi α-entropy and their comparison with those of κ entropy is provided that supports this line of inquiry [48].

*Renyi and* κ *entropy*: The presence of a logarithm function of the probability density in the expression of the von Neumann entropy −Tr(ρlnρ) implies that the entropy calculation requires the computation of the complete spectrum of the density matrix for its diagonalization, which can be computationally intensive for large systems. The Renyi entropy $\frac{1}{1-\alpha }\ln\left(Tr\rho^{\alpha }\right)$ instead involves a power of the probability density and is often computationally easier to estimate than the von Neumann entropy. As a result, the Renyi entropy is much more efficient and accessible for simulating complex quantum systems, as it relies on power traces rather than full eigenvalue decompositions of the von Neumann entropy, leading to faster and more scalable simulations.

The Renyi entropy is very flexible and depends on a free parameter that controls the entropy value. This allows more flexible forms and different weighting schemes of the probability distribution to be considered.

An important advantage of the variability of the free Renyi parameter is that by fixing it appropriately, the entropy measure can emphasize different aspects of the probability distribution of a quantum state by focusing on the most probable components or on the less dominant contributions to better understand the complex nature of the entanglement and quantum correlations. This versatility of the Renyi entropy can be particularly beneficial in the study of critical phenomena where certain entanglement features might be obscured by a single, fixed measure, or in phenomena where entanglement scaling and phase transitions reveal subtle quantum effects.

The Renyi entropy may be more accessible in experimental and computational settings than techniques such as interference-based measurements. The advantages of the Renyi entropy over the von Neumann entropy not only allow us to better characterize quantum states and gain deeper insights into the distribution and exchange of information between entangled particles, but also to improve error analysis and algorithm optimization in quantum computing.

The properties of the Renyi entropy, which make it faster to compute and more flexible than the von Neumann entropy, are due to its expression and, in particular, its dependence on a power of the probability density with a free parameter as the exponent. Of course, other entropies have also been considered in the literature, which share with the Renyi entropy the fact that they are also constructed from powers of the probability density and therefore share the main qualitative properties of the Renyi entropy. The choice of an entropy that generalizes the von Neumann entropy and uses powers instead of the logarithm of the probability density in its definition is very difficult and subjective.

Here, the κ-entropy is considered as a new paradigm of quantum entropy for two different reasons. The first is that the κ-entropy, like the Renyi entropy, is defined from the power of the probability density and thus shares the main qualitative features of the Renyi entropy. The second reason for choosing the κ-entropy, which makes it more interesting, is that it has a physical origin. The κ parameter of entropy has its roots in Einstein’s theory of special relativity. It is a relativistic generalization of the Boltzmann entropy of classical statistical mechanics and thus of the von Neumann quantum entropy. The κ-entropy can therefore be seen as the relativistic generalization of the von Neumann entropy, which results when the parameter κ approaches zero. The study of κ-entropy in the context of quantum computing and quantum information therefore allows us to consider quantum systems that have relativistic properties. This gives us a more comprehensive view of the nature of quantum-relativistic phenomena.

## 2. Kappa Entropy for Canonical States

The aim of this section is to consider a canonical Gibbs state density matrix for some Hamiltonian and determine an operational form for its kappa canonical quantum entropy via the expectation value of a quantum observable, Proposition 1; and further, to introduce a two-temperatures protocol for measuring the canonical state kappa entropy of a give quantum system, Proposition 2. Consider the following,

**Definition** **1.** *Let κ∈[0,1) and let the density $\rho \in D_{N}=\left\{\rho \in C^{N\times N};\rho^{†}=\rho ,\rho >0,Tr\rho =1\right\},$ the kappa entropy reads*

$$S_{\kappa }\left(\rho \right)=-\frac{1}{2\kappa }Tr\left(\rho^{\kappa +1}-\rho^{-\kappa +1}\right)$$

κ+1∈[1,2),−κ+1∈[1,0).

Next, we show how the canonical state kappa entropy is expressed via the expectation value of a quantum observable.

**Proposition** **1.** *The kappa entropy of a canonical state $\rho_{can}=\frac{1}{Z_{T}}e^{-\beta H},$$\beta =\frac{1}{kT},$ is cast in the form of an expectation value of the measurement of the quantum observable $C_{\kappa }=\frac{1}{\kappa }\sinh\left(\kappa \left(\ln Z_{T}I+\beta H\right)\right)$, in state $\rho_{can}$, i.e.,*

$$S_{\kappa }\left(\rho_{can}\right)=-Tr\left(\rho_{can}C_{\kappa }\right)=-\langle \rho_{can},C_{\kappa }\rangle .$$

*State and observable are commuting.*

Next, we put forward a two-temperatures protocol for measuring the canonical state kappa entropy,

**Proposition** **2.** *The kappa entropy of a canonical state $\rho_{can}=\frac{1}{Z_{T}}e^{-\beta H}$ of temperature T, and partition function $Z_{T}=Tr\left(e^{-\beta H}\right),$ is simulated by two quantum systems described by the same Hamiltonian each in the canonical Gibbs state of the respective κ-dependent temperatures $T_{cool}=\frac{T}{1+\kappa }<T$ (the cool system) and $T_{hot}=\frac{T}{1-\kappa }>T$ (the hot system), with corresponding partition functions $Z_{cool}\equiv Z_{\frac{T}{1+\kappa }}$ and $Z_{hot}\equiv Z_{\frac{T}{1-\kappa }}.$ The kappa entropy is expressed in terms of the partition functions of the simulating systems as*

(2) $$S_{\kappa }\left(\rho_{can}\right)=-\frac{1}{2\kappa }\frac{1}{Z_{T}}\left(Z_{\frac{T}{1+\kappa }}\times \frac{1}{Z_{T}^{\kappa }}-Z_{\frac{T}{1-\kappa }}\times Z_{T}^{\kappa }\right).$$

*The protocol*: For a given canonical density matrix $\rho_{can}$ with given Hamiltonian *H* and reference temperature $\beta =\frac{1}{kT}$, apply the following two-temperatures protocol in order to determine the kappa entropy $S_{\kappa }\left(\rho_{can}\right)$. Suppose the κ parameter is fixed and not adjustable, then introduce and control two temperatures: the high $T_{hot}\equiv T_{-\kappa }=\frac{T}{1-\kappa }$ and the low $T_{cool}\equiv T_{+\kappa }=\frac{T}{1+\kappa }$ temperatures, lying above and below the reference temperature *T*. By varying the high-T and the low-T temperature independently and determining the partition functions indicated in the last Equation (2), it is possible to simulate the value of the kappa entropy of the initial Gibbs state for any value of *T* and κ. Operationally, that would require letting two copies of the original system of temperature *T* interact with a heat bath that will increase its temperature T→$T_{hot}=\frac{T}{1-\kappa }$ in the first copy and decrease its temperature $T\to T_{cool}=\frac{T}{1+\kappa }$ in the second copy, and then form the combination of the partition function expressed in Equation (2).

## 3. Quantum Channels for κ Entropy

The aim of this section is to express the kappa entropy for a general density matrix via a positive and trace-preserving quantum channel as well as via its unitary dilation, Proposition 3 [49,50,51,52]; and further, to simulate the kappa entropy via a generalized form of the quantum circuit of the so-called Hadamard test, [53], Proposition 4. We introduce the following:

**Proposition** **3.** *By means of the formalism of the vectorization of matrices $A\to |A\rangle \rangle$ and the purification of the density matrices $\rho \to |\sqrt{\rho }\rangle \rangle ,$ the κ entropy $S_{\kappa }$ is expressed as*

$$S_{\kappa }\left(\rho \right)=Tr\left(Tr_{2}E_{\rho }\left(|I\rangle \rangle \langle \langle I|\right)\right),$$

*where for any $\nu \in C^{N\times N}⊗C^{N\times N}$, the positive semi-definite map $E_{\rho }:C^{N\times N}⊗C^{N\times N}\to C^{N\times N}⊗C^{N\times N},$ is introduced as, $E_{\rho }\left(\nu \right)=R_{+}\left(\rho \right)\nu R_{+}\left(\rho \right)-R_{-}\left(\rho \right)\nu R_{-}\left(\rho \right)$, with (Kraus-like) generators*

$$R_{\pm }\left(\rho \right)=\frac{1}{\sqrt{\kappa }}\left(\rho^{\frac{\pm \kappa +1}{2}}⊗I\right).$$

*Map $E_{\rho }$ is also expressed in an extended space, by adding an auxiliary qubit. The density matrix of the total, auxiliary+system, is defined on matrix space $\;C^{2\times 2}⊗C^{N\times N}⊗C^{N\times N}.$ Map $E_{\rho }$ is explicitly obtained as*

$$E_{\rho }\left(\nu \right)=Tr_{1}\left(U\left(\rho \right)\sigma_{3}⊗\nu U^{†}\left(\rho \right)\right)$$

*where the channel reads*

$$E_{\rho }\left(\nu \right)=Tr_{1}\left(\begin{array}{cc} \frac{1}{\sqrt{\kappa }}R_{+}\left(\rho \right)\\ \; & \frac{1}{\sqrt{\kappa }}R_{-}\left(\rho \right) \end{array}\right)\left(\begin{array}{cc} \nu \\ \; & -\nu \end{array}\right){\left(\begin{array}{cc} \frac{1}{\sqrt{\kappa }}R_{+}\left(\rho \right)\\ \; & \frac{1}{\sqrt{\kappa }}R_{-}\left(\rho \right) \end{array}\right)}^{†}.$$

*Kappa entropy via generalized Hadamard test circuit:*

Devise an operational construction which will enable the implementation of the transformation $\rho^{\pm \kappa }\to \rho^{1\pm \kappa },$ and further generate the quantity $S_{\kappa }$. To this end, next, we provide a quantum measurement procedure that evaluates the trace $Tr\rho^{1\pm \kappa },$ that is based on an extension to the density matrix formalism of the idea of the so-called ’Hadamard test’, initially used for pure states (see, e.g., [53]). The quantum circuit of the proposed measurement is given below.

Notation: AdX stands for the adjoint action of operator $X\in C^{N\times N},$ as follows $AdX\left(.\right)=X\left(.\right)X^{†}.$ E.g., let the control *X* gate $V_{cX}=P_{0}⊗X+P_{1}⊗I_{N},$ acting on the composite system with control on the qubit state and target on the reference system; the notation $AdV_{cX}$ means the adjoint action, i.e.,

$$AdV_{cX}\left(.\right)=V_{cX}\left(.\right)V_{cX}^{†}=\left(P_{0}⊗X+P_{1}⊗I\right)\left(.\right)\left(P_{0}⊗X^{†}+P_{1}⊗I\right).$$

**Figure 1:**
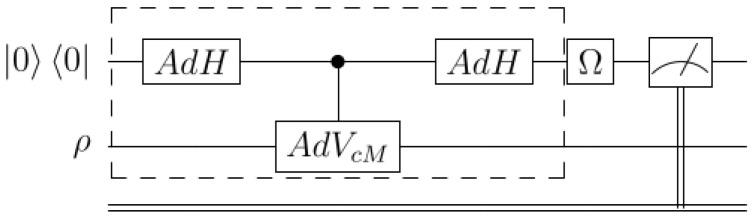
Quantum circuit of the map $T_{\Omega }\left[M\right]$ for generating the κ entropy of a density matrix state. The broken-line box represents the gate S[M], and the initial state is $|0\rangle \langle 0|⊗\rho$.

Simulation of kappa entropy via generalized Hadamard test quantum circuit (Proposition 4; c.f. Figure 1).

**Proposition** **4.** *Consider attaching to the Hilbert space $C^{N}$ of the reference quantum system, an auxiliary qubit so that the total state space is $H\approx C^{2}⊗C^{N}.$ Next, consider the initial state $|0\rangle \langle 0|⊗\rho ,$ where we denote by ρ the density matrix of the system for which we want to evaluate the kappa entropy. For a matrix $M\in End\left(C^{N}\right)\approx C^{N\times N},$ introduce the following map S[M]:D(H)→D(H) in the space of the density matrices $D\left(H\right)\approx D\left(C^{2}⊗C^{N}\right);$ explicitly, for H as the Hadamard matrix, the map S[M] is determined by composing the transformations $AdH⊗I_{N}$ and $AdV_{cM},$ where both of them operate on the composite system of reference+qubit, and reads,*

$$S\left[M\right]\equiv \left(AdH⊗I_{N}\right)\circ AdV_{cM}\circ \left(AdH⊗I_{N}\right).$$

*For *Ω *and M, operators on the auxiliary qubit and the reference systems, respectively, define the map $M\to T_{\Omega }\left[M\right],$*

$$T_{\Omega }\left[M\right]\equiv Tr_{1}\left\{\left(\Omega ⊗I_{N}\right)\circ S\left[M\right]\right\}:D\left(H\right)\to D\left(H\right),$$

*parametrized by some *Ω* and M. Acting on an initial state $|0\rangle \langle 0|⊗\rho ,$ map $T_{\Omega }\left[M\right]$ yields $T_{\pm \sigma_{3}}\left[\rho^{\pm \kappa }\right]=\pm \rho^{1\pm \kappa }.$ Denoting $T_{\pm \sigma_{3}}\equiv$$T_{\pm }$ the kappa entropy $S_{\kappa }\left(\rho \right)$ is generated acting on $|0\rangle \langle 0|⊗\rho$ as*

$$\begin{array}{cc} & Tr[\frac{1}{2\kappa }\left(T_{+}\left[\rho^{\kappa }\right]+T_{-}\left[\rho^{-\kappa }\right]\right)\left(|0\rangle \langle 0|⊗\rho \right)]\\ = & -\frac{1}{2\kappa }Tr\left[\left(\rho^{1+\kappa }-\rho^{1-\kappa }\right)\right]\\ = & S_{\kappa }\left(\rho \right).\;\;\;\;\;\;\;\;\;\; \end{array}$$

## 4. Set of Values and Bounds for κ Entropy

In this section, two questions are investigated: (i) What is the set of values of a (scaled) difference between the von Neumann and κ entropy (Proposition 6)?; and (ii) If an input density matrix is transformed by a unitary quantum channel, how is the output density matrix κ-entropy $S_{\kappa }$ affected? Explicit upper bounds on $S_{\kappa }$ are estimated that are determined by the stochastic properties of the channel generators, as well as by the spectral properties of the input-output density matrices, (Proposition 6).

**Proposition** **5.** *The κ quantum entropy within the validity of function $sinhc_{\pi }$ approximation, [54,55,56,57], is*

(3) $$S_{\kappa }\left(\rho \right)=S_{vN}\left(\rho \right)-\lim_{n\to \infty }Tr\left(\rho \ln\rho \sum_{m=1}^{n}\frac{1}{(2m+1)!}{\left(\pi \kappa \ln\rho \right)}^{2m}\right)$$

*so it reads as a κ-depended correction to the von Neumann quantum entropy.*

The following bounds are applied for the kappa entropy in terms of the vN entropy:

$$\begin{array}{ccc} S_{\kappa }\left(\rho \right) & \geq & S_{vN}\left(\rho \right)-\left(\lim_{n\to \infty }\frac{n}{(2n+1)!}\lambda_{\min}^{n}\right)\langle u_{\max}\left|\left(\Lambda \left|\ln\Lambda \right|\right)\right|u_{\max}\rangle \\ S_{\kappa }\left(\rho \right) & \leq & S_{vN}\left(\rho \right)-\left(\lim_{n\to \infty }\frac{n}{6}\lambda_{\max}\right)\langle u_{\max}|\left(\Lambda \left|\ln\Lambda \right|\right)\left|u_{\max}\rangle \right). \end{array}$$

Also, in terms of the maximal eigenvalue and eigenvector of $\left|\ln\rho \right|$ and by virtue of the Perron–Frobenius theorem, it is found that in the asymptotic limit (n→∞), the scaled difference of the two entropies has the interval of values:

$$\frac{S_{\kappa }\left(\rho \right)-S_{vN}\left(\rho \right)}{\langle u_{\max}|\left(\Lambda \left|\ln\Lambda \right|\right)\left|u_{\max}\rangle \right)}\in \left[0,\infty \right).$$

Bounded change of the kappa entropy due to quantum random unitary channel (Proposition 6) (see [58,59,60]):

**Proposition** **6.** *Let a quantum random unitary channel E that induces a transformation of $\rho \to \rho^{\prime }=E\left(\rho \right)=\sum_{i}p_{i}A_{i}\rho A_{i}^{†},$ with $A_{i}$ be its unitary generators and $p=(p_{i})$ the vector of its weights. Also, let $\lambda ,\lambda^{\prime }$ be the eigenvalue stochastic vectors of ρ and the $\rho^{\prime }$ density matrices, respectively. The transformed density matrix is used together with the channel to derive or estimate the κ-entropy. Its components $\rho^{\prime \pm \kappa +1}$ are bounded by bounds determined by the spectral parameters of the input density matrix, and by the spectral and stochastic parameters of the channel as follows:*

$$\begin{array}{ccc} Tr\rho^{\prime \kappa +1} & \leq & \eta_{\kappa }{\left(∥|p\rangle ∥∥|\lambda \rangle ∥\right)}^{\kappa +1},\\ Tr\rho^{\prime -\kappa +1} & \leq & \eta_{-\kappa }{\left(∥|p\rangle ∥∥|\lambda \rangle ∥\right)}^{-\kappa +1}. \end{array}$$

In the proof, the variable $\eta_{\kappa }=\sum_{i}{\left(\sqrt{TrH^{\left(i\right)}H^{(i)T}}\right)}^{\kappa +1}$ has been introduced, where the matrices $H_{jm}^{\left(i\right)}\equiv {\left(h^{j}\right)}_{im},$ and the circulant permutation $h|n\rangle =|n+1\rangle ,\mathrm{mod}N,$ have been used along with the following lemma [61,62,63,64]:

**Lemma** **1.** *Let the quantum random unitary channel transformation of $\rho \to \rho^{\prime }=E\left(\rho \right)=\sum_{i}p_{i}A_{i}\rho A_{i}^{†},$ with $A_{i}$ be unitary. Let $\lambda ,\lambda^{\prime }$ be the eigenvalue vectors of the ρ and $\rho^{\prime }$ density matrices, respectively, which are related by the unistochastic matrix $\Delta_{E}=\sum_{i}p_{i}A_{i}\circ A_{i}^{*},$ as $\lambda^{\prime }=\Delta_{E}\lambda .$ If, via Birkhoff’s theorem, the bi-stochastic matrix $\Delta_{E}$ is decomposed as a convex combination of circular permutations $\left\{h^{i}\right\},$ then $\Delta_{E}=\sum_{i}p_{i}h^{i}$.*

## 5. Summary and Outlook

The material covered in this paper provides tools and concepts from the field of quantum computation-information in order to enable a useful interaction with the theory of kappa entropy. The canonical state kappa entropy is shown to offer a framework where quantum computing would inspire operational methods for analyzing further the kappa entropy and motivate quantum mechanical measurements based on the entropy. Similarly, the quantum channel formalism and the constructive method of the Hadamard circuit that have been developed for κ entropy transformation and generation reveal an intimate relation of those techniques with entropy, and applications along those lines would be anticipated. Finally, the interrelations between the von Neumann entropy and κ entropy place the latter within the broad field of entropies, a fact that would enable the κ entropy to be applied in the fields of open quantum systems and master equations. Finally, we mention some specific research topics that the formalism put forward here could help investigate. Examples are as follows: (i) How does the κ entropy evolve in time for the density matrix of a qubit system evolving temporally by means of a time-dependent unitary evolution channel with a Kraus generator governed by a propagator determined by a coherent state path integral SU(2)∖SU(1,1)? [65]. (ii) How can the κ entropy quantify the entanglement developed among the coin system and the walker system in the course of diffusion of an anyonic quantum walk? [66]. (iii) A similar question to (i) but now with a Kraus generator governed by the Hamiltonian of a qubit that has developed a Berry adiabatic geometric phase in the framework of sudden-adiabatic approximation [67]. Also, unlike the three previous dynamic studies, a novel kinematical study for κ entropy is suggested. How does the κ entropy of a bipartite quantum system of a composite number dimension *d*, e.g., d=6, change if the parent system decomposes naturally based on the so-called prime-decomposition, and splits into two subsystems of dimension $d_{A}=2$ (qubit) and $d_{B}=3$ (qutrit)? [64]. Finally, addressing questions related to the relativistic origin and aspects of κ entropy, [4], is an interesting, open area that would benefit from the present formalism.

## Data Availability

No new data were created or analyzed in this study. Data sharing is not applicable to this article.

## Appendix A. Proofs

**Lemma** **A1** (Technical Lemma). *The various powers of the density matrices that are presented throughout the paper are all derived via the spectral decomposition (canonical decomposition) of the density matrix ρ. Recall that the density matrix in finite dimension $\rho \in D_{N}=\left\{\rho \in C^{N\times N};\rho^{†}=\rho ,\rho >0,Tr\rho =1\right\}$, where N≡[0,1,…,N−1], as well as its version in the infinite dimension $\rho \in D_{\infty }=\left\{\rho \in C^{N_{0}\times N_{0}};\rho^{†}=\rho ,\rho >0,Tr\rho =1\right\}.$ Given the solution of the eigenvalue/vector problem for ρ, its spectral decomposition follows as $\rho =U\Lambda U^{†}=\sum_{\lambda_{n}\in N}\lambda_{n}U\left|n\rangle \langle n\right|U^{†},$ where ρU=UΛ, with $\Lambda |n\rangle =\lambda_{n}|n\rangle ,$ the eigenvalues ${\left\{\lambda_{n}\right\}}_{n=0}^{N-1}$ with properties $\lambda_{n}\in \left[0,1\right],$$\sum_{n=0}^{N-1}\lambda_{n}=1;$ and $|u_{n}\rangle =U|n\rangle$ the corresponding eigenvectors ${\left\{|u_{n}\rangle \right\}}_{n=0}^{N-1}$ expressed in the canonical basis ${\left\{|n\rangle \right\}}_{n=0}^{N-1}$ forming an orthonormal and complete basis. Analogous properties are values for the infinite dimensional case $D_{\infty }$ where the set N is substituted by $N_{0}.$ Powers of the density matrix are evaluated following the properties of the decomposition, i.e., $\rho^{\kappa }=U\Lambda^{\kappa }U^{†}=\sum_{\lambda_{n}\in N}\lambda_{n}^{\kappa }\left|u_{n}\rangle \langle u_{n}\right|.$ A negative power would require ρ not to be singular and having all its eigenvalues non-zero, etc. [49].*

Let κ∈[0,1) and let the density $\rho \in D=\left\{\rho \in C^{N\times N};\rho^{†}=\rho ,\rho >0,Tr\rho =1\right\}$

**Proof of Proposition** **1.** Canonical distribution: Let $\rho^{1\pm \kappa }=e^{\left(1\pm \kappa \right)\ln\rho },$ and let the canonical Gibbs state $\rho_{can}=\frac{1}{Z}e^{-\beta H},$ with $Z=Tr(e^{-\beta H}).$ Then,

$$\ln\rho_{can}=\ln\frac{1}{Z}e^{-\beta H}=-\left(I\ln Z+\beta H\right).$$

Denote $\beta_{\pm \kappa }:=\left(1\pm \kappa \right)\beta$ and $Z_{\pm \kappa }:=Z^{1\pm \kappa }$; then similarly

$$\begin{array}{ccc} \rho_{can}^{1\pm \kappa } & = & e^{\left(1\pm \kappa \right)\ln\rho }=e^{-(1\pm \kappa )(I\ln Z+\beta H)}=e^{-(1\pm \kappa )\ln Z\;I}e^{-(1\pm \kappa )\beta H}\\ & = & \frac{1}{Z^{1\pm \kappa }}e^{-\left(1\pm \kappa \right)\beta H}, \end{array}$$

Proceed now to a quantum measurement determination of the kappa entropy: Let $\rho^{1\pm \kappa }=\rho \rho^{\pm \kappa }.$ Assume $\rho =\rho_{can}=\frac{1}{Z}e^{-\beta H},$ then

$$\begin{array}{ccc} \rho_{can}^{1\pm \kappa } & = & \rho_{can}\rho_{can}^{\pm \kappa }=\frac{1}{Z}e^{-\beta H}\frac{1}{Z^{\pm \kappa }}e^{-(\pm \kappa )\beta H}\\ & = & \rho_{can}\frac{1}{Z^{\pm \kappa }}e^{-(\pm \kappa )\beta H} \end{array}$$

Computing the kappa entropy for the canonical state yields

$$\begin{array}{ccc} S_{\kappa }\left(\rho_{can}\right) & = & -\frac{1}{2\kappa }Tr\left(\rho_{can}^{1+\kappa }-\rho_{can}^{1-\kappa }\right)\\ & = & -\frac{1}{2\kappa }Tr(\rho_{can}\left(\rho_{can}^{\kappa }-\rho_{can}^{-\kappa }\right) \end{array}$$

and further

$$\begin{array}{cc} S_{\kappa }\left(\rho_{can}\right) & =\frac{1}{2\kappa }Tr\left(\frac{1}{Z}e^{-\beta H}\left(\frac{1}{Z^{-\kappa }}e^{\kappa \beta H}-\frac{1}{Z^{\kappa }}e^{-\kappa \beta H}\right)\right)\\ & =\frac{1}{2\kappa }Tr\left(\frac{1}{Z}e^{-\beta H}\left(\left[\frac{1}{Z^{-\kappa }}\cosh\left(\kappa \beta H\right)-\frac{1}{Z^{\kappa }}\cosh\left(\kappa \beta H\right)\right]+\right.\right.\\ & \;\left.\left.\left[\frac{1}{Z^{-\kappa }}\sinh\left(\kappa \beta H\right)+\frac{1}{Z^{\kappa }}\sinh\left(\kappa \beta H\right)\right]\right)\right). \end{array}$$

This leads to

$$S_{\kappa }\left(\rho_{can}\right)=\frac{1}{2\kappa }Tr\left\{\frac{1}{Z}e^{-\beta H}\left[\left(\frac{1}{Z^{-\kappa }}-\frac{1}{Z^{\kappa }}\right)\cosh\left(\kappa \beta H\right)+\left(\frac{1}{Z^{\kappa }}+\frac{1}{Z^{-\kappa }}\right)\sinh\left(\kappa \beta H\right)\right]\right\}.$$

By means of the expression $\frac{1}{2}\left(\frac{1}{Z^{-\kappa }}-\frac{1}{Z^{\kappa }}\right)=\sinh\left(\kappa \ln Z\right),$ and $\frac{1}{2}\left(\frac{1}{Z^{-\kappa }}+\frac{1}{Z^{\kappa }}\right)=\cosh\left(\kappa \ln Z\right),$ we compute

$$S_{\kappa }\left(\rho_{can}\right)=\frac{1}{\kappa }Tr\left\{\rho_{can}\left[\sinh(\kappa \ln Z)\cosh(\kappa \beta H)+\cosh(\kappa \ln Z)\sinh(\kappa \beta H)\right]\right\},$$

and thanks to the identity sinh(x)cosh(y)+cosh(x)sinh(y)=sinh(x+y), we obtain

(A1) $$S_{\kappa }\left(\rho_{can}\right)=Tr\left[\rho_{can}\cdot \frac{1}{\kappa }\sinh\left(\kappa I\ln Z+\kappa \beta H\right)\right].$$

□

**Proof of Proposition** **2.** Two-temperatures simulation protocol of kappa entropy. Always 1±κ>0, so let $\left(1\pm \kappa \right)\beta =\underset{︸}{\left(1\pm \kappa \right)\frac{1}{kT}}=\frac{1}{k\left(\frac{T}{1\pm \kappa }\right)}=\underset{︸}{\frac{1}{kT_{\pm \kappa }}},$ define the *effective temperatures*

$$\begin{array}{ccc} T_{cool} & \equiv & T_{+\kappa }=\frac{T}{1+\kappa }<T\;,\;\;\\ T_{hot} & \equiv & T_{-\kappa }=\frac{T}{1-\kappa }\geq T\;. \end{array}$$

So, compute the power $\rho_{can}^{1\pm \kappa }$

$$\begin{array}{cc} \rho_{can}^{1\pm \kappa } & =\frac{1}{Z^{1\pm \kappa }}e^{-\frac{1\pm \kappa }{kT}H}\\ & =\frac{1}{Z^{1\pm \kappa }}e^{-\frac{1}{kT_{\pm \kappa }}H}. \end{array}$$

The kappa entropy reads

$$\begin{array}{cc} S_{\kappa }\left(\rho_{can}\right) & =-\frac{1}{2\kappa }Tr\left(\rho_{can}^{1+\kappa }-\rho_{can}^{1-\kappa }\right)\\ & =-\frac{1}{2\kappa }Tr\left(\frac{1}{Z^{1+\kappa }}e^{-\frac{1}{kT_{+\kappa }}H}-\frac{1}{Z^{1-\kappa }}e^{-\frac{1}{kT_{-\kappa }}H}\right) \end{array}$$

or

$$S_{\kappa }\left(\rho_{can}\right)=:-\frac{1}{2\kappa }Tr\left(\frac{Z_{cool}}{Z^{1+\kappa }}\cdot \rho_{cool}-\frac{Z_{hot}}{Z^{1-\kappa }}\cdot \rho_{hot}\right),$$

where the two auxiliary canonical density matrices are

$$\begin{array}{ccc} \rho_{cool} & = & \frac{1}{Z_{cool}}e^{-\frac{1}{kT_{cool}}H}\;;Z_{cool}=Tre^{-\frac{1}{kT_{cool}}H}\\ \rho_{hot} & = & \frac{1}{Z_{hot}}e^{-\frac{1}{kT_{hot}}H};Z_{hot}=Tre^{-\frac{1}{kT_{hot}}H}. \end{array}$$

Due to normalization $Tr\rho_{hot}=Tr\rho_{cool}=1,$ the following is true

$$\begin{array}{cc} S_{\kappa }\left(\rho_{can}\right) & =-\frac{1}{2\kappa }\left(\frac{Z_{cool}}{Z^{1+\kappa }}\cdot Tr\rho_{cool}-\frac{Z_{hot}}{Z^{1-\kappa }}\cdot Tr\rho_{hot}\right)\\ & =-\frac{1}{2\kappa }\left(\frac{Z_{cool}}{Z^{1+\kappa }}-\frac{Z_{hot}}{Z^{1-\kappa }}\right)=-\frac{1}{2\kappa }\frac{Z_{cool}Z^{1-\kappa }-Z_{hot}Z^{1+\kappa }}{Z^{1+\kappa }Z^{1-\kappa }}\\ & =-\frac{1}{2\kappa }\frac{Z_{cool}Z^{1-\kappa }-Z_{hot}Z^{1+\kappa }}{Z^{2}} \end{array}$$

or

$$\begin{array}{cc} S_{\kappa }\left(\rho_{can}\right) & =-\frac{1}{2\kappa }\left(Z_{cool}Z^{-1-\kappa }-Z_{hot}Z^{-1+\kappa }\right)\\ & =-\frac{1}{2\kappa }\left(Z_{\frac{T}{1+\kappa }}Z^{-1-\kappa }-Z_{\frac{T}{1-\kappa }}Z^{-1+\kappa }\right)\;. \end{array}$$

To emphasize, denote *Z* by $Z_{T},$ so

$$\begin{array}{ccc} S_{\kappa }\left(\rho_{can}\right) & = & -\frac{1}{2\kappa }\left(Z_{\frac{T}{1+\kappa }}Z_{T}^{-1-\kappa }-Z_{\frac{T}{1-\kappa }}Z_{T}^{-1+\kappa }\right)\;\;\;\;\\ \;\;S_{\kappa }\left(\rho_{can}\right) & = & -\frac{1}{2\kappa }\left(Z_{cool}Z^{-1-\kappa }-Z_{hot}Z^{-1+\kappa }\right)\\ \;\;\;\;\;S_{\kappa }\left(\rho_{can}\right) & = & -\frac{1}{2\kappa Z}\left(\frac{Z_{cool}}{Z^{\kappa }}-Z_{hot}Z^{\kappa }\right). \end{array}$$

□

*Protocol*: cooling, heating, post-processing

Example: let κ=2/3.

Compute $T\to T_{cool}=\frac{T}{1+\kappa }=\frac{3}{5}T$ and evaluate $Z_{cool}$,

Compute $T\to T_{hot}=\frac{T}{1-\kappa }=3T$ and evaluate $Z_{hot}$,

Compute (post-processing) $S_{\kappa }\left(\rho_{can}\right)$ in terms of $Z,Z_{cool}\left(\kappa \right),Z_{hot}\left(\kappa \right)$ and κ.

These steps can be repeated for any κ.

**Proof of Proposition** **3.** *Preliminaries*: *The “double-wedge” notation*: Any bipartite quantum system may be described by the state vector

$$|\psi \rangle =\sum_{i=1}^{n_{1}}\sum_{j=1}^{n_{2}}c_{ij}|\phi_{i}\rangle ⊗|x_{j}\rangle ,$$

with $\sum_{i=1}^{n_{1}}\sum_{j=1}^{n_{2}}{\left|c_{ij}\right|}^{2}=1$. Consider the following state vector $|\psi \rangle$ of two qubits in $\left\{|0\rangle ,|1\rangle \right\}$:

$$|\psi \rangle =c_{00}|00\rangle +c_{01}|01\rangle +c_{10}|10\rangle +c_{11}|11\rangle =\left(\begin{array}{cc} |0\rangle & |1\rangle \end{array}\right)\left(\begin{array}{cc} c_{00} & c_{01}\\ c_{10} & c_{11} \end{array}\right)\left(\begin{array}{c} |0\rangle \\ |1\rangle \end{array}\right)$$

There is an equivalent expression for bipartite systems called the “double-wedge” ket vector $|A\rangle \rangle$. In particular, *A* is the matrix representation of a quantum system $|\psi \rangle$, whose element in position (i,j) is the respective coefficient $c_{ij}$ of $|\psi \rangle$. So, in this case:

$$|A\rangle \rangle =|\left(\begin{array}{cc} A_{00} & A_{01}\\ A_{10} & A_{11} \end{array}\right)\rangle \rangle$$

Due to this description, a double-wedge ket $|A\rangle \rangle$ must also be normalized as a prerequisite of the preservation of the probability constraint, meaning that ${∥|A\rangle \rangle ∥}_{2}={∥A∥}_{F}=1$, where the Euclidean vector norm equals the Frobenius matrix norm, i.e., ${∥|A\rangle \rangle ∥}_{2}=\sqrt{\langle \langle A||A\rangle \rangle }=\sqrt{Tr(A^{†}A)}={∥A∥}_{F}$. Moreover, the following local transformation A→(V⊗W)A, where the V,W are unitaries, preserves normalization as well as the partial traces of the bipartite projector $|A\rangle \rangle \langle \langle A|$ (see below). This “double-wedge” notation exploits the isomorphism between vectors in $H_{1}⊗H_{2}$ and the rectangular $n_{1}\cdot n_{2}$ matrices, where $n_{1}$ and $n_{2}$ are the dimensions of $H_{1}$ and $H_{2}$, respectively. Due to the isomorphism, the matrices $A\in C^{n_{1}\times n_{2}}$ and vectors $|A\rangle \rangle \in C^{n_{1}\cdot n_{2}}$, are expressed as follows:

$$A=\sum_{i=1}^{n_{1}}\sum_{j=1}^{n_{2}}A_{ij}|i\rangle \langle j|↔|A\rangle \rangle =\sum_{i=1}^{n_{1}}\sum_{j=1}^{n_{2}}A_{ij}|i\rangle ⊗|j\rangle$$

Similar to the vector representation, there is also a “double-wedge” bra vector:

$$\langle \langle A|={\left(|A\rangle \rangle \right)}^{†}=\langle \langle \left(\begin{array}{cc} A_{00}^{*} & A_{01}^{*}\\ A_{10}^{*} & A_{11}^{*} \end{array}\right)|$$

Some notable properties of the double-wedge ket notation include the following:

$$\begin{array}{ccc} \left(A⊗C^{T}\right)|B\rangle \rangle & = & |ABC\rangle \rangle \\ \left(A⊗I\right)|B\rangle \rangle & = & |AB\rangle \rangle \\ \left(I⊗C^{T}\right)|B\rangle \rangle & = & |AC\rangle \rangle \\ {∥|A\rangle \rangle ∥}_{2} & = & \sqrt{\langle \langle A||A\rangle \rangle }=\sqrt{Tr(A^{†}A)}={∥A∥}_{F}\\ \langle \langle A||B\rangle \rangle & = & Tr(A^{†}B)\\ |A+B\rangle \rangle & = & |A\rangle \rangle +|B\rangle \rangle \end{array}$$

Several notable examples of double-wedge state vectors and matrices exist such that the Bell states and Pauli matrices are presented as follows:

$$\begin{array}{ccc} |X\rangle \rangle & = & |\frac{1}{\sqrt{2}}\left(\begin{array}{cc} 0 & 1\\ 1 & 0 \end{array}\right)\rangle \rangle =\frac{1}{\sqrt{2}}(|01\rangle +\left|10\rangle \right)\\ |iY\rangle \rangle & = & |\frac{i}{\sqrt{2}}\left(\begin{array}{cc} 0 & -i\\ i & 0 \end{array}\right)\rangle \rangle =\frac{1}{\sqrt{2}}(|01\rangle -\left|10\rangle \right) \end{array}$$

$$\begin{array}{ccc} |Z\rangle \rangle & = & |\frac{1}{\sqrt{2}}\left(\begin{array}{cc} 1 & 0\\ 0 & -1 \end{array}\right)\rangle \rangle =\frac{1}{\sqrt{2}}(|00\rangle -\left|11\rangle \right)\\ |I\rangle \rangle & = & |\frac{1}{\sqrt{2}}\left(\begin{array}{cc} 1 & 0\\ 0 & 1 \end{array}\right)\rangle \rangle =\frac{1}{\sqrt{2}}(|00\rangle +|11\rangle ). \end{array}$$

Considering that a bipartite system is in a pure state, its respective density matrix ρ is equal to $|\psi \rangle \langle \psi |$, which is equivalent to $|A\rangle \rangle \langle \langle A|$ with reference to its double-wedge ket notation. In addition, the calculation of the reduced density matrices is performed according to the following formulas:

$$\begin{array}{ccc} Tr_{2}\left(|A\rangle \rangle \langle \langle A|\right) & = & AA^{†}\\ Tr_{1}\left(|A\rangle \rangle \langle \langle A|\right) & = & A^{T}A^{*} \end{array}$$

and

$$\langle \langle A||B\rangle \rangle =Tr\left(A^{†}B\right).$$

*Purified mixed-state kappa entropy*: Consider ρ∈D and the map $\rho \to |\sqrt{\rho }\rangle \rangle .$ The reduced systems obtained from the bipartite system $|\sqrt{\rho }\rangle \rangle$ by partial tracing one of each subsystem are the original single partite density matrix, i.e.,

$$\begin{array}{ccc} Tr_{1}|\sqrt{\rho }\rangle \rangle \langle \langle \sqrt{\rho }| & = & \sqrt{\rho }{\sqrt{\rho }}^{†}=\rho \\ Tr_{2}|\sqrt{\rho }\rangle \rangle \langle \langle \sqrt{\rho }| & = & {\sqrt{\rho }}^{T}{\sqrt{\rho }}^{*}=\rho . \end{array}$$

Purification is non-unique since the map $\rho \to W\rho W^{†}$ of the initial mixed state by a unitary operator *W* leads to the same reduce density matrix, i.e., $\sqrt{\rho }=\sqrt{W\rho W^{†}}=W\sqrt{\rho }W^{†}$

$$\begin{array}{ccc} Tr_{1}|W\sqrt{\rho }W^{†}\rangle \rangle \langle \langle W\sqrt{\rho }W^{†}| & = & W\sqrt{\rho }W^{†}W\sqrt{\rho }W^{†}=W\rho W^{†}\\ Tr_{2}|W\sqrt{\rho }W^{†}\rangle \rangle \langle \langle W\sqrt{\rho }W^{†}| & = & {\left(W\sqrt{\rho }W^{†}\right)}^{T}{\left(W\sqrt{\rho }W\right)}^{*}=W\rho W^{†}. \end{array}$$

Further, consider the spectral decomposition of the density matrix $\rho =VDV^{†},$ then $\sqrt{\rho }=V\sqrt{D}V^{†},$ and also ${\sqrt{\rho }}^{*}=V^{*}\sqrt{D}V^{†*},$ then $\sqrt{\rho }{\sqrt{\rho }}^{†}=\left(V\sqrt{D}V^{†}\right)\left(V^{*}\sqrt{D}V^{†*}\right).$ Next, elaborate on the purification map $\rho \to |V\sqrt{D}V^{†}\rangle \rangle$ to obtain

$$\begin{array}{cc} |\sqrt{\rho }\rangle \rangle & =|V\sqrt{D}V^{†}\rangle \rangle =V⊗V^{*}|\sqrt{D}\rangle \rangle \\ & =\left(V⊗V^{*}\right)\sum_{i=1}^{s}\sqrt{p_{i}}|i\rangle ⊗|i\rangle . \end{array}$$

The 1-norm reads ${∥|\sqrt{\rho }\rangle \rangle ∥}_{1}={∥|\sqrt{D}\rangle \rangle ∥}_{1}=\sum_{i=1}^{s}\sqrt{p_{i}},$ where Sch $\left(|\sqrt{\rho }\rangle \rangle \right)=s$ is the Schmidt number. Next, we give various equivalent forms of expressing the density matrices of mixed quantum systems via their associated (non-unique) pure state vectors of some bipartite system: first

$$\rho \to |\sqrt{\rho }\rangle \rangle =\sqrt{N}\sqrt{\rho }⊗I|\frac{1}{\sqrt{N}}I\rangle \rangle =\sqrt{\rho }⊗I|I\rangle \rangle =I⊗{\sqrt{\rho }}^{T}|I\rangle \rangle ,$$

and also

$$\begin{array}{cc} \rho & \to |\rho \sqrt{\rho }\rangle \rangle =\sqrt{N}\rho \sqrt{\rho }⊗I|\frac{1}{\sqrt{N}}I\rangle \rangle \\ & =\sqrt{\rho }⊗\rho^{T}|I\rangle \rangle \\ & =\rho ⊗{\sqrt{\rho }}^{T}|I\rangle \rangle =I⊗\rho^{T}{\sqrt{\rho }}^{T}|I\rangle \rangle \\ & =I⊗{\sqrt{\rho }}^{T}\rho^{T}|I\rangle \rangle . \end{array}$$

*Main proof*: Utilizing the identity $Tr_{2}\left(|A\rangle \rangle \langle \langle A|\right)=AA^{†}$ for the case

$$Tr_{2}|\rho^{\frac{\kappa +1}{2}}\rangle \rangle \langle \langle \rho^{\frac{\kappa +1}{2}}|=\rho^{\kappa +1},$$

the following expression for the kappa entropy is obtained:

$$\begin{array}{cc} S_{\kappa }\left(\rho \right) & =-\frac{1}{\kappa }Tr[Tr_{2}\left(|\rho^{\frac{\kappa +1}{2}}\rangle \rangle \langle \langle \rho^{\frac{\kappa +1}{2}}|-|\rho^{\frac{-\kappa +1}{2}}\rangle \rangle \langle \langle \rho^{\frac{-\kappa +1}{2}}|\right)\\ & =-\frac{1}{\kappa }Tr_{2}\left(\rho^{\frac{\kappa +1}{2}}⊗I|I\rangle \rangle \langle \langle I|\rho^{\frac{\kappa +1}{2}}⊗I-\rho^{\frac{-\kappa +1}{2}}⊗I|I\rangle \rangle \langle \langle I|\rho^{\frac{-\kappa +1}{2}}⊗I\right)]. \end{array}$$

The $S_{\kappa }$ is expressed as

$$S_{\kappa }\left(\rho \right)=Tr_{2}E\left(|I\rangle \rangle \langle \langle I|\right),$$

where for any $\nu \in C^{N\times N}⊗C^{N\times N},$ the positive semi-definite map $E:C^{N\times N}⊗C^{N\times N}\to C^{N\times N}⊗C^{N\times N}$ is introduced,

$$E\left(\nu \right)=R_{+}\nu R_{+}-R_{-}\nu R_{-}$$

with (Kraus-like) generators

$$R_{\pm }=\frac{1}{\sqrt{\kappa }}\left(\rho^{\frac{\pm \kappa +1}{2}}⊗I\right).$$

Via the relation $\rho^{\frac{\pm \kappa +1}{2}}=e^{\frac{\pm \kappa +1}{2}\ln\rho }$, the generators are also expressed in the form

$$\begin{array}{cc} R_{\pm } & =\frac{1}{\sqrt{\kappa }}e^{\frac{\pm \kappa +1}{2}\ln\rho ⊗I}\\ & =\frac{1}{\sqrt{\kappa }}\left(\sqrt{\rho }⊗I\right)e^{\frac{\pm \kappa }{2}\ln\rho ⊗I}. \end{array}$$

Further, map E is also expressed in an extended space by adding an auxiliary qubit. The density matrix of the total (auxiliary+reference) system is defined on matrix space $\;C^{2\times 2}⊗C^{N\times N}⊗C^{N\times N}.$ Map E is explicitly obtained as

$$E\left(\nu \right)=Tr_{1}\left(U\sigma_{3}⊗\nu U^{†}\right)$$

where $\sigma_{3}$ is the state given to the auxiliary qubit and *U* is a conditional gate with generators $\rho^{\frac{\pm \kappa +1}{2}}$

$$\begin{array}{cc} U & =\left(\begin{array}{cc} \frac{1}{\sqrt{\kappa }}R_{+} & \\ & \frac{1}{\sqrt{\kappa }}R_{-} \end{array}\right)=\left(\begin{array}{cc} \frac{1}{\sqrt{\kappa }}\rho^{\frac{\kappa +1}{2}} & \\ & \frac{1}{\sqrt{\kappa }}\rho^{\frac{-\kappa +1}{2}} \end{array}\right)\\ & =\frac{1}{\sqrt{\kappa }}\left(P_{0}⊗R_{+}+P_{1}⊗R_{-}\right), \end{array}$$

which reads explicitly

$$\begin{array}{cc} U & =\frac{1}{\sqrt{\kappa }}\left(I⊗\sqrt{\rho }⊗I\right)\left(\begin{array}{cc} e^{\frac{\kappa }{2}\ln\rho ⊗I} & \\ & e^{\frac{-\kappa }{2}\ln\rho ⊗I} \end{array}\right)\\ & =\frac{1}{\sqrt{\kappa }}\left(I⊗\sqrt{\rho }⊗I\right)e^{\frac{\kappa }{2}\sigma_{3}⊗\ln\rho ⊗I}. \end{array}$$

So, the channel reads

$$E\left(\nu \right)=Tr_{1}\left(\begin{array}{cc} \frac{1}{\sqrt{\kappa }}R_{+}\\ \; & \frac{1}{\sqrt{\kappa }}R_{-} \end{array}\right)\left(\begin{array}{cc} \nu \\ \; & -\nu \end{array}\right){\left(\begin{array}{cc} \frac{1}{\sqrt{\kappa }}R_{+}\\ \; & \frac{1}{\sqrt{\kappa }}R_{-} \end{array}\right)}^{†}$$

□

*Addendum*

$S_{\kappa =\frac{1}{2}}$ *via purification:*

Utilizing this possibility of expressing a mixed state as a reduction of its associated pure states, we express the kappa entropy $S_{\kappa }$ for $\kappa =\frac{1}{2}$ as

$$\begin{array}{cc} S_{\kappa =\frac{1}{2}}\left(\rho \right) & =2\left({∥|\sqrt{\rho }\rangle \rangle ∥}_{1}-{∥|\rho \sqrt{\rho }\rangle \rangle ∥}_{1}\right)\\ & =2(\sum_{i=1}^{N}{\left(p_{i}\right)}^{1/2}-\sum_{i=1}^{N}{\left(p_{i}\right)}^{3/2}). \end{array}$$

This result is valid for any density matrix ρ up to a unitary transformation, i.e.,

$$\rho \to W\rho W^{†}\Rightarrow \rho \to \sqrt{\rho }=\sqrt{W\rho W^{†}}=W\sqrt{\rho }W^{†}=\sqrt{WVDV^{†}W^{†}}=WV\sqrt{D}{\left(WV\right)}^{†}$$

then

$$\rho \to |\sqrt{\rho }\rangle \rangle =|\sqrt{W\rho W^{†}}\rangle \rangle =WV⊗{\left(WV\right)}^{*}|\sqrt{D}\rangle \rangle ,$$

due the local unitary invariance of the 1-norm,

$${∥|\sqrt{W\rho W^{†}}\rangle \rangle ∥}_{1}={∥|\sqrt{\rho }\rangle \rangle ∥}_{1},$$

so, $S_{\kappa =\frac{1}{2}}\left(W\rho W^{†}\right)=S_{\kappa =\frac{1}{2}}\left(\rho \right).$

Recall next the triangular inequality for the $l_{1}$ norm, ${∥x∥}_{1}+{∥y∥}_{1}\leq {∥x+y∥}_{1},$$\left|{∥x∥}_{1}-{∥y∥}_{1}\right|\leq {∥x-y∥}_{1},$ and applying it to the last equation leads to an upper bound for the kappa entropy, this time determined only by the spectrum of the related density matrix; it reads

$$\begin{array}{cc} \left|S_{\kappa =\frac{1}{2}}\left(\rho \right)\right| & =2\left|{∥|\sqrt{\rho }\rangle \rangle ∥}_{1}-{∥|\rho \sqrt{\rho }\rangle \rangle ∥}_{1}\right|\leq 2{∥|\sqrt{\rho }-\rho \sqrt{\rho }\rangle \rangle ∥}_{1}\\ & \leq 2\sum_{i=1}^{N}\left({\left(p_{i}\right)}^{1/2}-{\left(p_{i}\right)}^{3/2}\right).\;\;\;\;\;\;\;\;\;\;\;\;\;\;\;\;\;\;\;\;\;\;\;\;\;\;\;\;\;\;\;\;\;\;\;\;\;\;\;\;\;\;\;\;\;\;\; \end{array}$$

**Proof of Proposition** **4.** Consider attaching to the Hilbert space $C^{N}$ of the reference quantum system, an auxiliary qubit so the total state space is $H\approx C^{2}⊗C^{N}.$ Next, consider the initial state $|0\rangle \langle 0|⊗\rho ,$ where we denote with ρ the density matrix of the system for which we want to evaluate the kappa entropy. Introduce the following map on the density matrices S[M]:D(H)→D(H) explicitly composed of maps acting on the composite system of reference+qubits,

$$S\left[M\right]\equiv AdH⊗I\circ AdV_{cM}\circ AdH⊗I:D\left(H\right)\to D\left(H\right),$$

on an initial state, i.e., $|0\rangle \langle 0|⊗\rho \to S\left[M\right]\left(|0\rangle \langle 0|⊗\rho \right),$

$$|0\rangle \langle 0|⊗\rho \overset{AdH⊗I}{\to }\;\overset{AdV_{cM}}{\to }\;\overset{AdH⊗I}{\to }\;|0\rangle \langle 0|⊗\rho \;$$

where the adjoint action $A\to XAX^{†}$ has been denoted as A→AdX(A). Specifically, AdH⊗I stands for the local adjoint action of the Hadamard gate *H* on the auxiliary qubit state, i.e., $Ad\left(H⊗I\right)\rho_{2}⊗\rho_{N}=H\rho_{2}H^{†}⊗\rho_{N}$. Also, $AdV_{cM}$ stands for the control *M* gate $V_{cM}=P_{0}⊗M+P_{1}⊗I,$ acting on the composite system with control on the qubit state and the target on the reference system.

$$AdV_{cM}\left(.\right)=V_{cM}\left(.\right)V_{cM}^{†}=\left(P_{0}⊗M+P_{1}⊗I\right)\left(.\right)\left(P_{0}⊗M^{†}+P_{1}⊗I\right)$$

Next, let $M:C^{N}\to C^{N}$ as the matrix introduced before and also $\Omega :C^{2}\to C^{2}$ for a matrix acting on an the auxiliary qubit space. Define map $T_{\Omega }\left[M\right],$

$$T_{\Omega }\left[M\right]\equiv Tr_{1}\left(\Omega ⊗I\right)\circ S\left[M\right]:D\left(H\right)\to D\left(H\right),$$

parametrized by some Ω and M. Map T describes the action taken after S[M] on the total auxiliary qubit+reference density matrix state, e.g., $|0\rangle \langle 0|⊗\rho .$ On the resulting state $S\left[M\right]\left(|0\rangle \langle 0|⊗\rho \right)$, the mean value of the qubit space operator Ω is measured next, i.e., $Tr_{1}\left(\Omega ⊗I\right)S\left[M\right](|0\rangle \langle 0|⊗\rho .$ *Examples*: the choice Ω=I leads to

$$Tr_{1}\left(\Omega ⊗I\right)S\left[M\right]\left(|0\rangle \langle 0|⊗\rho \right)=\frac{1}{2}\left(M\rho M^{†}+\rho \right),$$

the choice $\Omega =|0\rangle \langle 0|-|1\rangle \langle 1|\equiv \sigma_{3}$ leads to

$$Tr_{1}\left(\Omega ⊗I\right)S\left[M\right]\left(|0\rangle \langle 0|⊗\rho \right)=\frac{1}{2}\left(\rho M^{†}+M\rho \right).$$

Also, if M=ρ, then $S\left[\rho \right]\left(|0\rangle \langle 0|⊗\rho \right)=\rho^{2}.$ The choices $M=\rho^{\pm \kappa },$ then give

$$S\left[\rho^{\pm \kappa }\right]\left(|0\rangle \langle 0|⊗\rho \right)=\rho^{1\pm \kappa }.$$

In explicitly matrix form:

$$\begin{array}{cc} & S\left[M\right]\left(\left(\begin{array}{cc} \rho & .\\ . & . \end{array}\right)\right)\\ = & \frac{1}{4}\underset{︸}{\left(\begin{array}{cc} I & I\\ I & -I \end{array}\right)\left(\begin{array}{cc} M & .\\ . & I \end{array}\right)\left(\begin{array}{cc} I & I\\ I & -I \end{array}\right)}\left(\begin{array}{cc} \rho & .\\ . & . \end{array}\right)\underset{︸}{\left(\begin{array}{cc} I & I\\ I & -I \end{array}\right)\left(\begin{array}{cc} M^{†} & .\\ . & I \end{array}\right)\left(\begin{array}{cc} I & I\\ I & -I \end{array}\right)}\\ = & \frac{1}{4}\underset{︸}{\left(\begin{array}{cc} M+I & M-I\\ M-I & M+I \end{array}\right)}\left(\begin{array}{cc} \rho & .\\ . & . \end{array}\right)\underset{︸}{\left(\begin{array}{cc} M^{†}+I & M^{†}-I\\ M^{†}-I & M^{†}+I \end{array}\right)}\\ = & \frac{1}{4}\left(\begin{array}{cc} \rho \left(M^{†}+I\right)+M\rho \left(M^{†}+I\right) & M\rho \left(M^{†}+I\right)-\rho \left(M^{†}+I\right)\\ \rho \left(M^{†}-I\right)+M\rho \left(M^{†}-I\right) & M\rho \left(M^{†}-I\right)-\rho \left(M^{†}-I\right) \end{array}\right). \end{array}$$

The choice $\Omega =\sigma_{3},$ i.e., $\Omega ⊗I=\left(\begin{array}{cc} I & \\ & -I \end{array}\right)$ leads to

$$\begin{array}{cc} & Tr_{1}\left(\left(\Omega ⊗I\right)S\left[M\right]\left(\left(\begin{array}{cc} \rho & .\\ . & . \end{array}\right)\right)\right)\\ = & Tr_{1}\frac{1}{4}\left(\begin{array}{c} I\\ -I \end{array}\right)\left(\begin{array}{cc} \rho \left(M^{†}+I\right)+M\rho \left(M^{†}+I\right) & M\rho \left(M^{†}+I\right)-\rho \left(M^{†}+I\right)\\ \rho \left(M^{†}-I\right)+M\rho \left(M^{†}-I\right) & M\rho \left(M^{†}-I\right)-\rho \left(M^{†}-I\right) \end{array}\right)\\ = & \frac{1}{4}Tr_{1}\left(\begin{array}{cc} \rho \left(M^{†}+I\right)+M\rho \left(M^{†}+I\right) & M\rho \left(M^{†}+I\right)-\rho \left(M^{†}+I\right)\\ -\rho \left(M^{†}-I\right)-M\rho \left(M^{†}-I\right) & -M\rho \left(M^{†}-I\right)+\rho \left(M^{†}-I\right) \end{array}\right). \end{array}$$

Which yields

$$\begin{array}{cc} & Tr_{1}\left(\left(\begin{array}{c} I\\ -I \end{array}\right)S\left[M\right]\left(\left(\begin{array}{cc} \rho & .\\ . & . \end{array}\right)\right)\right)\\ = & \frac{1}{4}\left\{\left(\rho \left(M^{†}+I\right)+M\rho \left(M^{†}+I\right)-M\rho \left(M^{†}-I\right)+\rho \left(M^{†}-I\right)\right)\right\}\\ = & \frac{1}{4}\left\{\rho M^{†}+\rho +M\rho M^{†}+M\rho -M\rho M^{†}+M\rho +\rho M^{†}-\rho \right\}\\ = & \frac{1}{2}\left(\rho M^{†}+M\rho \right).\;\;\;\;\;\;\; \end{array}$$

Moreover,

$$\begin{array}{ccc} Tr_{1}\left(\left(\sigma_{3}⊗I_{N}\right)S\left[\rho^{\kappa }\right]\left(\left(\begin{array}{cc} \rho & .\\ . & . \end{array}\right)\right)\right) & = & \rho^{1+\kappa },\\ Tr_{1}\left(\left(-\sigma_{3}⊗I_{N}\right)S\left[\rho^{-\kappa }\right]\left(\left(\begin{array}{cc} \rho & .\\ . & . \end{array}\right)\right)\right) & = & -\rho^{1-\kappa }. \end{array}$$

So, $T_{\pm \sigma_{3}}\left[\rho^{\pm \kappa }\right]=\pm \rho^{1\pm \kappa }.$ Abbreviating $T_{\pm \sigma_{3}}$ to $T_{\pm }$ yields the kappa entropy

$$-Tr\frac{1}{2\kappa }\left(T_{+}\left[\rho^{\kappa }\right]+T_{-}\left[\rho^{-\kappa }\right]\right)\left(|0\rangle \langle 0|⊗\rho \right)=-Tr\frac{1}{2\kappa }\left(\rho^{1+\kappa }-\rho^{1-\kappa }\right)=S_{\kappa }\left(\rho \right).$$

□

**Proof of Proposition** **5.** The following work-flow outlines the content of the proof.

$\begin{array}{cccc} S_{\kappa }\left(\rho \right) & \begin{array}{c} ↗\\ ↘ \end{array} & \begin{array}{c} \sin\mathrm{hc}\;\mathrm{modulation}\\ \sin\mathrm{hc}\;\mathrm{approximation} \end{array} & \begin{array}{c} \to \\ \to \end{array}\begin{array}{c} S_{\kappa }\left(\rho \right)∼\sin h\;c\left(\rho \right)S_{vN}\left(\rho \right)\\ S_{\kappa }\left(\rho \right)=S_{vN}\left(\rho \right)-\lim_{n\to \infty }Tr\left(\rho \left|\ln\rho \right|{\hat{R}}_{n}\right) \end{array}\\ & & & \begin{array}{c} ↓\\ \mathrm{PF}\text{-}\mathrm{Thm}\\ ↓\\ \mathrm{what}’s\;\lim_{n\to \infty }{\hat{R}}_{n}\\ ↓\\ \frac{1}{\nu }\left(S_{\kappa }\left(\rho \right)-S_{vN}\left(\rho \right)\right)\in \left[0,\infty \right) \end{array} \end{array}$

The proof starts with the κ entropy $S_{\kappa }$ of a density matrix $\rho =U\Lambda U^{†}$, and shows, by employing the sinhc function, that it admits two decompostions; one *multiplicative*, in which $S_{\kappa }$ is shown to equal a von Neumann entropy $S_{vN},$ that is modulated with sinhc as the modulation kernel function, and one *additive*, in which $S_{\kappa }$ equals $S_{vN}$ plus an operator limiting function. This limiting function is shown to be computed by invoking the Perron–Frobenius theorem for positive matrices, that involve the operator $\left|\ln\Lambda \right|.$ Finally, the κ weighted difference of $S_{\kappa }$ and $S_{vN}$ entropies is shown to be non-negative real. Let $\rho =\sum_{i=1}^{N}p_{i}|u_{i}\rangle \langle u_{i}|=U\Lambda U^{†}$ be the spectral decomposition of the density matrix. Consider the real powers of the density matrix

$$\begin{array}{ccc} \rho^{\pm \kappa +1} & = & U\Lambda^{\pm \kappa +1}U^{†}=U\Lambda^{\pm \kappa }U^{†}\\ & = & \sum_{i}p_{i}^{\pm \kappa }|u_{i}\rangle \langle u_{i}|, \end{array}$$

where $p_{i}^{\pm \kappa }:=e^{\pm \kappa \ln p_{i}}.$ Define the hyperbolic cardinal sine function [54],

$$sinhc\left(x\right)=\left\{\begin{array}{cc} 1 & \mathrm{for}\;x=0\\ \frac{\sinh x}{x} & \mathrm{for}\;x\in R\setminus \left\{0\right\}\;\mathrm{or}\;x\in C\setminus \left\{0\right\} \end{array}\right.$$

or its normalized form

$$sinhc_{\pi }\left(x\right)=\left\{\begin{array}{cc} 1 & \mathrm{for}\;x=0\\ \frac{\sinh\pi x}{\pi x} & \mathrm{for}\;x\in R\setminus \left\{0\right\}\;\mathrm{or}\;x\in C\setminus \left\{0\right\} \end{array}\right.$$

where $\sinh x=\frac{1}{2}\left(e^{x}-e^{-x}\right).$ Then, (assuming $p_{i}\neq 0$ for all *i*’s), the spectral decomposition of the kappa entropy reads

(A2) $$\begin{array}{ccc} S_{\kappa }\left(\rho \right) & = & -\frac{1}{2\kappa }Tr\left(\rho^{\kappa +1}-\rho^{-\kappa +1}\right)=-\frac{1}{2\kappa }Tr\left(\rho \left(\rho^{\kappa }-\rho^{-\kappa }\right)\right)\\ & = & -\frac{1}{\kappa }Tr\left(\rho \left(\sum_{i}\sinh\left(\kappa \ln p_{i}\right)|u_{i}\rangle \langle u_{i}|\right)\right)\\ & = & -Tr(\sum_{i}p_{i}\ln p_{i}\underset{︸}{\frac{\sinh(\kappa \ln p_{i})}{\kappa \ln p_{i}}}|u_{i}\rangle \langle u_{i}|) \end{array}$$

rearranging

$$\begin{array}{ccc} S_{\kappa }\left(\rho \right) & = & -\frac{1}{\kappa }Tr\left(\sum_{i}p_{i}\times \kappa \ln p_{i}\;sinhc\left(\kappa \ln p_{i}\right)|u_{i}\rangle \langle u_{i}|\right),\\ S_{\kappa }\left(\rho \right) & = & -\frac{1}{\kappa }Tr\sum_{i}p_{i}\left(\ln p_{i}^{\kappa }\;sinhc\left(\ln p_{i}^{\kappa }\right)\right)|u_{i}\rangle \langle u_{i}|. \end{array}$$

*Remark*: The above Equation (A2), $S_{\kappa }\left(\rho \right)=TrS_{vN}\left(\rho \right)sinhc\left(\kappa \ln\rho^{\kappa }\right)$ shows that the κ-entropy of a density operator ρ is expressed as a self-modulation of its von Neumann entropy $S_{vN}\left(\rho \right)$ by a kernel function identified with the sinhc function with the argument depending on $\kappa \ln p_{i},$ i.e., $sinhc(\kappa \ln p_{i}),$ in the eigenbasis of the ρ operator. This property provides a direct relation between $S_{\kappa }$ and $S_{vN}.$ Treating $S_{\kappa }$ as $S_{vN}$ with sinhc modulation for any discrete probability distribution function (d-pdf) would suggest another application. The κ entropy could be applied when studying the sequences of d-pdf’s $p^{1}≻p^{2}⊁p^{3}≻\cdots ≻p^{n-1}≻p^{n}$ that violate the majorization condition ≻ ([68]), and exhibit an evolution to an equilibrium state where the entropy is allowed to decrease locally, while it increases globally in the asymptotic limit. Such a phenomenon appears in the study of the evolution of a quantum walk ([69]). The evolution of d-pdf in that case, due to the braking of majorization ordering, exhibits a modulation of an otherwise monotonic sequence of entropy values in small subsets, c.f. Figure 7, and related discussion ([69]). Next, we proceed to introduce two approximations in the above equation for $S_{\kappa }\left(\rho \right)$: first, denote by *x* the sequence, $x={\left(x_{i}\right)}_{i}\equiv {\left(\ln p_{i}^{\kappa }\right)}_{i}$ to cast the kappa entropy in the form

(A3) $$S_{\kappa }\left(\rho \right)=-\frac{1}{\kappa }Tr\sum_{i}p_{i}(x_{i}\;\left(sinhc\left(x_{i}\right)\right)|u_{i}\rangle \langle u_{i}|.$$

Recall the necessary numerical-algebraic background: The Chebyshev–Stirling numbers of both kinds are known in the literature ([55,56,57]) as the case $\gamma =\frac{1}{2}$ of the Jacobi–Stirling numbers of both kinds determined by means of the recurrence relations

$$\;{\left[\begin{array}{c} n\\ k \end{array}\right]}_{\gamma }=\;{\left[\begin{array}{c} n-1\\ k-1 \end{array}\right]}_{\gamma }+\left(n-1\right)\left(n+2\gamma -2\right)\;{\left[\begin{array}{c} n-1\\ k \end{array}\right]}_{\gamma }$$

and

$$\;{\left\{\begin{array}{c} n\\ k \end{array}\right\}}_{\gamma }=\;{\left\{\begin{array}{c} n-1\\ k-1 \end{array}\right\}}_{\gamma }+k\left(k+2\gamma -1\right)\;{\left\{\begin{array}{c} n-1\\ k \end{array}\right\}}_{\gamma }$$

with initial conditions

$$\;{\left[\begin{array}{c} n\\ 0 \end{array}\right]}_{\gamma }=\;{\left\{\begin{array}{c} n\\ 0 \end{array}\right\}}_{\gamma }=\delta_{0n}\;\;\mathrm{and}\;\;{\left[\begin{array}{c} 0\\ k \end{array}\right]}_{\gamma }=\;{\left\{\begin{array}{c} 0\\ k \end{array}\right\}}_{\gamma }=\delta_{0k}.$$

We proceed with the following two approximations of the function sinhc in terms of the Jacobi–Stirling (JS) numbers ${\left[\begin{array}{c} a\\ b \end{array}\right]}_{m}.$ *Approximation 1*: an asymptotic formula for the Chebyshev–Stirling numbers of the first kind ([56])

$$\begin{array}{ccc} sinhc_{\pi }\left(x\right) & ∼ & \frac{1}{{\left(n!\right)}^{2}}\sum_{m=0}^{n}{\left[\begin{array}{c} n+1\\ m+1 \end{array}\right]}_{1/2}x^{2m},n\to \infty \\ x\sin hc_{\pi }\left(x\right) & ∼ & \frac{1}{{\left(n!\right)}^{2}}\sum_{m=0}^{n}{\left[\begin{array}{c} n+1\\ m+1 \end{array}\right]}_{1/2}x^{2m+1},n\to \infty . \end{array}$$

A second approximation of the JS numbers is valid: *Approximation 2*: For m=0,…,n, ([57])

$$\frac{1}{{\left(n!\right)}^{2}}{\left[\begin{array}{c} n+1\\ m+1 \end{array}\right]}_{1/2}∼\frac{\pi^{2m}}{(2m+1)!},n\to \infty .$$

Combining approximations 1 and 2 yields the following expansion:

$$x\sin hc_{\pi }\left(x\right)∼\sum_{m=0}^{n}\frac{\pi^{2m}}{(2m+1)!}x^{2m+1},n\to \infty .$$

Back to the kappa entropy

$$S_{\kappa }\left(\rho \right)=-\lim_{n\to \infty }Tr\sum_{i=1}^{N}\sum_{m=0}^{n}\frac{{\left(\pi \kappa \right)}^{2m}}{(2m+1)!}p_{i}{\left(\ln p_{i}\right)}^{2m+1}|u_{i}\rangle \langle u_{i}|.$$

which is now expressed as

$$S_{\kappa }\left(\rho \right)=-\lim_{n\to \infty }Tr\sum_{m=0}^{n}\frac{{\left(\pi \kappa \right)}^{2m}}{(2m+1)!}\rho {\left(\ln\rho \right)}^{2m+1},$$

or in terms of the vN entropy $S_{vN}\left(\rho \right)=-Tr\left(\rho \ln\rho \right),$ equivalently as

(A4) $$S_{\kappa }\left(\rho \right)=S_{vN}\left(\rho \right)-\lim_{n\to \infty }Tr\sum_{m=1}^{n}\frac{{\left(\pi \kappa \right)}^{2m}}{(2m+1)!}\rho {\left(\ln\rho \right)}^{2m+1}.\;\;\;\;\;\;\;\;\;\;\;\;\;\;$$

We turn next to the evaluation of the bounds: first, c.f. the spectral decompositions $\rho =U\Lambda U^{†},\Lambda =\sum_{i=1}^{M}p_{i}\left|u_{i}\rangle \langle u_{i}\right|,$ and

$$\begin{array}{ccc} \rho {\left(\ln\rho \right)}^{2m+1} & = & \sum_{i=1}^{N}p_{i}{\left(\ln p_{i}\right)}^{2m+1}\left|u_{i}\rangle \langle u_{i}\right|\\ & = & U\left(\Lambda {\left(\ln\Lambda \right)}^{2m+1}\right)U^{†} \end{array}$$

and observe that since $0<p_{i}<1,$ then $\ln p_{i}$ is negative real, i.e., $\ln p_{i}=-\left|\ln p_{i}\right|,$ so ${\left(\ln p_{i}\right)}^{2m+1}={\left(-\left(\left|\ln p_{i}\right|\right)\right)}^{2m+1}=-{\left|\ln p_{i}\right|}^{2m+1}.$ Then,

$$\begin{array}{ccc} S_{\kappa }\left(\rho \right) & = & S_{vN}\left(\rho \right)+\lim_{n\to \infty }Tr\sum_{i=1}^{N}\sum_{m=1}^{n}\frac{{\left(\pi \kappa \right)}^{2m}}{(2m+1)!}p_{i}{\left|\ln p_{i}\right|}^{2m+1}|u_{i}\rangle \langle u_{i}|,\\ S_{\kappa }\left(\rho \right) & = & S_{vN}\left(\rho \right)+\lim_{n\to \infty }Tr\sum_{i=1}^{N}p_{i}\left|\ln p_{i}\right|\left(\sum_{m=1}^{n}\frac{1}{(2m+1)!}{\left(\pi \kappa \left|\ln p_{i}\right|\right)}^{2m}\right)|u_{i}\rangle \langle u_{i}|. \end{array}$$

Then, by means of the eigen-projectors $P_{i}=|u_{i}\rangle \langle u_{i}|$ and the defining property $P_{i}=P_{i}^{2}=P_{i}P_{j}\delta_{ij}$, we obtain that

(A5) $$S_{\kappa }\left(\rho \right)=S_{vN}\left(\rho \right)+\lim_{n\to \infty }Tr\left\{\left(\rho \left|\ln\rho \right|\right)\sum_{m=1}^{n}\frac{1}{(2m+1)!}{\left(\pi \kappa \left|\ln\rho \right|\right)}^{2m}\right\}.$$

*Bounds*: Next, bounds will be worked out for the second term in the rhs of the last equation above. First, we invoke the content of the Perron–Frobenius theorem. The bounds to be worked out will be evaluated by means of the relations

(A6) $$\begin{array}{ccc} \frac{1}{(2n+1)!}\sum_{m=1}^{n}{\left(\pi \kappa \left|\ln p_{i}\right|\right)}^{2m} & \leq & \sum_{m=1}^{n}\frac{1}{(2m+1)!}{\left(\pi \kappa \left|\ln p_{i}\right|\right)}^{2m}\leq \frac{1}{(2+1)!}\sum_{m=1}^{n}{\left(\pi \kappa \left|\ln p_{i}\right|\right)}^{2m}\\ & & \mathrm{or}\;\\ \frac{n}{(2n+1)!}\underset{︸}{\frac{1}{n}\sum_{m=1}^{n}{\left(\pi \kappa \left|\ln p_{i}\right|\right)}^{2m}} & \leq & \sum_{m=1}^{n}\frac{1}{(2m+1)!}{\left(\pi \kappa \left|\ln p_{i}\right|\right)}^{2m}\leq \frac{n}{6}\underset{︸}{\frac{1}{n}\sum_{m=1}^{n}{\left(\pi \kappa \left|\ln p_{i}\right|\right)}^{2m}}. \end{array}$$

The special case of matrix A, by means of the previous assumptions and the properties: (i) $\lambda_{\max}^{1}$ is the largest of all powers of $\lambda_{\max}$ and $\lambda_{\min};$ (ii) $\lambda_{\min}^{n}$ is the smallest of all powers of $\lambda_{\max}$ and $\lambda_{\min},$ yields the bounds

$$\begin{array}{ccc} \frac{n}{(2n+1)!}\underset{︸}{\frac{1}{n}\sum_{m=1}^{n}\lambda_{\max}^{m}{\left(\frac{{\left(\pi \kappa \left|\ln p_{i}\right|\right)}^{2}}{\lambda_{\max}}\right)}^{m}} & \leq & \sum_{m=1}^{n}\frac{1}{(2m+1)!}{\left(\pi \kappa \left|\ln p_{i}\right|\right)}^{2m} \end{array}$$

(A7) $$\begin{array}{ccc} & \leq & \frac{n}{6}\underset{︸}{\frac{1}{n}\sum_{m=1}^{n}\lambda_{\max}^{m}{\left(\frac{{\left(\pi \kappa \left|\ln p_{i}\right|\right)}^{2}}{\lambda_{\max}}\right)}^{m}}\\ & & \mathrm{or}\\ \frac{n}{(2n+1)!}\lambda_{\min}^{n}\underset{︸}{\frac{1}{n}\sum_{m=1}^{n}{\left(\frac{{\left(\pi \kappa \left|\ln p_{i}\right|\right)}^{2}}{\lambda_{\max}}\right)}^{m}} & \leq & \sum_{m=1}^{n}\frac{1}{(2m+1)!}{\left(\pi \kappa \left|\ln p_{i}\right|\right)}^{2m} \end{array}$$

(A8) $$\begin{array}{c} \;\;\;\;\;\;\;\;\;\;\;\leq \frac{n}{6}\lambda_{\max}\underset{︸}{\frac{1}{n}\sum_{m=1}^{n}{\left(\frac{{\left(\pi \kappa \left|\ln p_{i}\right|\right)}^{2}}{\lambda_{\max}}\right)}^{m}} \end{array}$$

Next, we work out the lower and the upper limit of the concerned sum

$$Tr\left\{\left(\rho \left|\ln\rho \right|\right)\lim_{n\to \infty }\sum_{m=1}^{n}\frac{1}{(2m+1)!}{\left(\pi \kappa \left|\ln\rho \right|\right)}^{2m}\right\},$$

by means of PF-Thm, and complete the proof by showing that the set of values of the scaled difference of κ and the vN entropy is the interval [0,∞). *Perron–Frobenius theorem*: Let the positive matrix $A={\left(A_{ij}\right)}_{i,j=1}^{N}$ with $A_{ij}>0.$ Let the simple eigenvalues of *A* ordered as $\left|\lambda_{1}\right|\geq \left|\lambda_{2}\right|\geq \cdots \geq \left|\lambda_{N}\right|.$ Denote $\lambda_{\min}=\lambda_{N},$ and by $\lambda_{\max}\equiv \lambda_{1}<1$, the spectral radius or maximal or dominant eigenvalue, and let $|u_{\max}\rangle$ be its associated eigenvector. (1) *Power limit*: Then, it is valid that

$$\lim_{n\to \infty }{\left(\frac{A}{\lambda_{\max}}\right)}^{n}=\left|u_{\max}\rangle \langle u_{\max}\right|.$$

(2) *Cesaro summation limit*: With a matrix *A* as above, it is valid that

$$\lim_{n\to \infty }\frac{1}{n}\sum_{m=1}^{n}{\left(\frac{A}{\lambda_{\max}}\right)}^{m}=\left|u_{\max}\rangle \langle u_{\max}\right|.$$

Below the PF-Thm, the special case of the matrix $A\equiv {\left(\pi \kappa \left|\ln\Lambda \right|\right)}^{2},$ where the eigenvalues of the ρ density matrix form a discrete probability distribution, will be denoted by $p_{i},i=1,\ldots ,N,$ and also where the matrix $A^{\prime }s$ maximal eigenvalue $\lambda_{\max}$ will be assumed to be $0<\lambda_{\max}\leq 1$. *Lower bound*: For the rhs of Equation (A5), we obtain via the inequalities of Equation (A6)

$$\begin{array}{cc} & Tr\left\{\left(\rho \left|\ln\rho \right|\right)\lim_{n\to \infty }\sum_{m=1}^{n}\frac{1}{(2m+1)!}{\left(\pi \kappa \left|\ln\rho \right|\right)}^{2m}\right\}\\ \geq & \left(\underset{n\to \infty }{\;\lim}\frac{n}{(2n+1)!}\frac{n}{(2n+1)!}\lambda_{\min}^{n}\right)Tr\left(\left(\rho \left|\ln\rho \right|\right)\underset{\hat{R}}{\underset{︸}{\lim_{n\to \infty }\frac{1}{n}\sum_{m=1}^{n}{\left(\frac{{\left(\pi \kappa \left|\ln\rho \right|\right)}^{2}}{\lambda_{\max}}\right)}^{m}}}\right). \end{array}$$

Next, the evaluation of the operator limit $\hat{R}\equiv \lim_{n\to \infty }\frac{1}{n}\sum_{m=1}^{n}{\left(\frac{{\left(\pi \kappa \left|\ln\rho \right|\right)}^{2}}{\lambda_{\max}}\right)}^{m}$ will be based on the Perron-Frobenius theorem (PF-Thm) in one of its equivalent expressions that involves the so-called Cesaro summation ([49,51,70]). Applying the PF-Thm in its Cesaro summation limit for matrix $A\equiv {\left(\pi \kappa \left|\ln\rho \right|\right)}^{2}$ yields

$$\begin{array}{ccc} & & Tr\left(\left(\rho \left|\ln\rho \right|\right)\underset{R}{\cdot \;\underset{︸}{\lim_{n\to \infty }\frac{1}{n}\sum_{m=1}^{n}{\left(\frac{{\left(\pi \kappa \left|\ln\rho \right|\right)}^{2}}{\lambda_{\max}}\right)}^{m}}}\right)\\ & = & Tr(\left(\rho \left|\ln\rho \right|\right)\cdot |u_{\max}\rangle \langle u_{\max}|)\\ & = & \langle u_{\max}\left|\left(\Lambda \left|\ln\Lambda \right|\right)\right|u_{\max}\rangle . \end{array}$$

Then, due to the limit $\underset{n\to \infty }{\;\lim}\frac{n}{(2n+1)!}=0,$ the previous evaluation yields the lower bound

(A9) $$\begin{array}{cc} & Tr\left\{\left(\rho \left|\ln\rho \right|\right)\lim_{n\to \infty }\sum_{m=1}^{n}\frac{1}{(2m+1)!}{\left(\pi \kappa \left|\ln\rho \right|\right)}^{2m}\right\}\\ \geq & \left(\underset{n\to \infty }{\lim}\frac{n}{(2n+1)!}\lambda_{\min}^{n}\right)\langle u_{\max}\left|\left(\Lambda \left|\ln\Lambda \right|\right)\right|u_{\max}\rangle . \end{array}$$

*Upper bound*: Next, recalling again Equation (A6), consider the sum

(A10) $$\begin{array}{cc} & Tr\left\{\left(\rho \left|\ln\rho \right|\right)\lim_{n\to \infty }\sum_{m=1}^{n}\frac{1}{(2m+1)!}{\left(\pi \kappa \left|\ln\rho \right|\right)}^{2m}\right\}\\ \leq & \left(\lim_{n\to \infty }\frac{n}{(2+1)!}\right)\lambda_{\max}Tr\left(\left(\rho \left|\ln\rho \right|\right)\underset{︸}{\lim_{n\to \infty }\frac{1}{n}\sum_{m=1}^{n}\frac{{\left(\pi \kappa \left|\ln\rho \right|\right)}^{2m}}{\lambda_{\max}}}\right)\\ \leq & \left(\lim_{n\to \infty }\frac{n}{6}\right)\lambda_{\max}Tr(\left(\Lambda \left|\ln\Lambda \right|\right)|u_{\max}\rangle \langle u_{\max}\left|\right)\\ \leq & \left(\lim_{n\to \infty }\frac{n}{6}\right)\lambda_{\max}\langle u_{\max}\left|\left(\Lambda \left|\ln\Lambda \right|\right)\right|u_{\max}\rangle . \end{array}$$

Referring to Equation (A4), and combining the last inequalities in Equations (A9) and (A10), we obtain that $S_{\kappa }\left(\rho \right)$ is bounded as

$$\begin{array}{ccc} S_{\kappa }\left(\rho \right) & \geq & S_{vN}\left(\rho \right)+\left(\lim_{n\to \infty }\frac{n}{(2n+1)!}\lambda_{\min}^{n}\right)\langle u_{\max}\left|\left(\Lambda \left|\ln\Lambda \right|\right)\right|u_{\max}\rangle \\ \;\;\;S_{\kappa }\left(\rho \right) & \leq & S_{vN}\left(\rho \right)+\left(\lim_{n\to \infty }\frac{n}{6}\lambda_{\max}\right)\langle u_{\max}|\left(\Lambda \left|\ln\Lambda \right|\right)\left|u_{\max}\rangle \right). \end{array}$$

Elaborating on the bounds of the scaled difference between the κ and von Neumann entropies leads to

$$\left(\lim_{n\to \infty }\frac{n}{(2n+1)!}\lambda_{\min}^{n}\right)\leq \frac{S_{\kappa }\left(\rho \right)-S_{vN}\left(\rho \right)}{\langle u_{\max}|\left(\Lambda \left|\ln\Lambda \right|\right)\left|u_{\max}\rangle \right)}\leq \left(\lim_{n\to \infty }\frac{n}{6}\lambda_{\max}\right).$$

Finally, the asymptotic interval of values of the scaled difference of the two entropies becomes

(A11) $$\begin{array}{ccc} \frac{S_{\kappa }\left(\rho \right)-S_{vN}\left(\rho \right)}{\langle u_{\max}|\left(\Lambda \left|\ln\Lambda \right|\right)\left|u_{\max}\rangle \right)} & \in & \left[\lim_{n\to \infty }\frac{n}{(2n+1)!}\lambda_{\min}^{n},\lim_{n\to \infty }\frac{n}{6}\lambda_{\max}\right)\\ & \in & [0,\infty ). \end{array}$$

□

**Proof of Proposition** **6.** Let the trace of the transformed density matrix, and recall from the previous proposition, be $\lambda^{\prime }=\Delta_{E}\lambda ,$ where $\Delta_{E}=\sum_{i}p_{i}A_{i}\circ A_{i}^{*},$ then

$$Tr\rho^{\prime \kappa +1}=Tr\Lambda^{\prime \kappa +1}=\sum_{i}\lambda_{i}^{\prime \kappa +1}=\sum_{i}{\left({\left(\Delta_{E}\lambda \right)}_{i}\right)}^{\kappa +1}=\sum_{i}{\left(\underset{︸}{\sum_{j}p_{j}{\left(h^{j}\lambda \right)}_{i}}\right)}^{\kappa +1}.$$

Elaborate the last sum

$$\begin{array}{ccc} \sum_{j}p_{j}{\left(h^{j}\lambda \right)}_{i} & = & \sum_{j}p_{j}\sum_{m}{\left(h^{j}\right)}_{im}\lambda_{m}=\sum_{j}\sum_{m}p_{j}{\left(h^{j}\right)}_{im}\lambda_{m}\\ & = & \sum_{j}\sum_{m}p_{j}H_{jm}^{\left(i\right)}\lambda_{m}=p^{T}H^{\left(i\right)}\lambda \end{array}$$

where $H_{jm}^{\left(i\right)}\equiv {\left(h^{j}\right)}_{im},$ and $H=\sum_{i}H^{\left(i\right)}.$ Returning to the trace expression

$$\begin{array}{ccc} Tr\rho^{\prime \kappa +1} & = & \sum_{i}{\left(p^{T}H^{\left(i\right)}\lambda \right)}^{\kappa +1}=\sum_{i}{\left(TrH^{\left(i\right)}\lambda p^{T}\right)}^{\kappa +1}=\sum_{i}{\left(\langle p|H^{\left(i\right)}|\lambda \rangle \right)}^{\kappa +1}\\ & = & \sum_{i}(Tr{\left(H^{\left(i\right)}|\lambda \rangle \langle p|\right)}^{\kappa +1} \end{array}$$

where $|\lambda \rangle =\sum_{i=0}^{N-1}\lambda_{i}|i\rangle ,$ and $|p\rangle =\sum_{i=0}^{N-1}p_{i}|i\rangle .$ Next, we look for an upper bound to the last sum above. Since

$$\begin{array}{ccc} \langle p|H^{\left(i\right)}|\lambda \rangle & = & Tr\left(H^{\left(i\right)}|\lambda \rangle \langle p|\right)\leq \sqrt{TrH^{\left(i\right)}H^{{\left(i\right)}^{T}}}\sqrt{Tr|\lambda \rangle \langle p|{\left(|\lambda \rangle \langle p|\right)}^{T}}\\ & = & \sqrt{TrH^{\left(i\right)}H^{{\left(i\right)}^{T}}}\sqrt{Tr|\lambda \rangle \langle p||p\rangle \langle \lambda |}\\ & = & \sqrt{TrH^{\left(i\right)}H^{{\left(i\right)}^{T}}}\sqrt{Tr|\lambda \rangle \langle p||p\rangle \langle \lambda |}\\ & = & \sqrt{TrH^{\left(i\right)}H^{(i)T}}\sqrt{\langle p|p\rangle \langle \lambda |\lambda \rangle } \end{array}$$

we find

$$Tr\rho^{\prime \kappa +1}\leq \sum_{i}{\left(\sqrt{TrH^{\left(i\right)}H^{(i)T}}\right)}^{\kappa +1}{\left(\sqrt{\langle p|p\rangle \langle \lambda |\lambda \rangle }\right)}^{\kappa +1}=\eta_{\kappa }{\left(\sqrt{\langle p|p\rangle \langle \lambda |\lambda \rangle }\right)}^{\kappa +1}$$

where $\eta_{\kappa }=\sum_{i}{\left(\sqrt{TrH^{\left(i\right)}H^{(i)T}}\right)}^{\kappa +1}.$ Summarizing

$$\begin{array}{ccc} Tr\rho^{\prime \kappa +1} & \leq & \eta_{\kappa }{\left(∥|p\rangle ∥∥|\lambda \rangle ∥\right)}^{\kappa +1}\\ Tr\rho^{\prime -\kappa +1} & \leq & \eta_{-\kappa }{\left(∥|p\rangle ∥∥|\lambda \rangle ∥\right)}^{-\kappa +1} \end{array}$$

Comment on the result obtained. The constants are expressed in terms of the matrix norms of the *h* elements,

$$\begin{array}{ccc} \eta_{\kappa } & = & \sum_{i}{\left(\sqrt{TrH^{\left(i\right)}H^{(i)T}}\right)}^{\kappa +1}=\Sigma_{i}{∥H^{\left(i\right)}∥}^{\kappa +1}\\ \eta_{-\kappa } & = & \Sigma_{i}{∥H^{\left(i\right)}∥}^{-\kappa +1} \end{array}$$

□

**Proof of** **Lemma.** Let $\rho^{\prime }=U\Lambda^{\prime }U^{†},\rho =V\Lambda V^{†},$ the canonical decomposition of the density matrices with $\Lambda^{\prime }=diag\left(\lambda^{\prime }\right)$ and Λ=diag(λ). Schematically,

$\begin{array}{ccc} \rho & \overset{E}{\to } & \rho^{\prime }\\ ↓ & ↓\\ V\Lambda V^{†} & \to & U\Lambda^{\prime }U^{†}\\ ↓ & ↓\\ \Lambda =diag(\lambda ) & \to & \Lambda^{\prime }=diag\left(\lambda^{\prime }\right)\\ ↓ & ↓\\ \lambda & \underset{\Delta_{E}}{\to } & \lambda^{\prime }=\Delta_{E}\lambda \end{array}$

Here, $h=\sum_{i=0}^{N-1}|i+1\rangle \langle i|$, where i+1 is compute mod N, the generator of circulant matrices $\left\{h^{0}=h^{N}=I,h^{1},h^{2},\ldots ,h^{N-1}\right\}.$ It follows that $\lambda^{\prime }=\Delta_{E}\lambda ,$ where $\Delta_{E}=\sum_{i}p_{i}A_{i}\circ A_{i}^{*}.$ □
